# Integrating digital and in-person therapy for PTSD: feasibility and acceptability of blended trauma-focused cognitive therapy in routine care

**DOI:** 10.3389/fpsyt.2024.1447651

**Published:** 2024-09-05

**Authors:** Johan Lundin, Markus Jansson-Fröjmark, Linda Gustafsson-Björverud, Nick Grey, Fredrik Santoft, Anke Ehlers, Per Carlbring, Tobias Lundgren, Maria Bragesjö, Sigrid Salomonsson

**Affiliations:** ^1^ Department of Clinical Neuroscience, Centre for Psychiatry Research, Karolinska Institute and Stockholm Health Care Services, Stockholm, Sweden; ^2^ Sussex Partnership NHS Foundation Trust and School of Psychology, University of Sussex, Brighton, United Kingdom; ^3^ Department of Experimental Psychology, University of Oxford, Oxford, United Kingdom; ^4^ Department of Psychology, Stockholm University, Stockholm, Sweden

**Keywords:** posttraumatic stress disorder, blended trauma-focused cognitive therapy, blended treatment, internet-based treatment program, routine care, trauma-focused cognitive behavioural therapy

## Abstract

**Introduction:**

Access to evidence-based psychological therapy for posttraumatic stress disorder (PTSD) is limited. Blended Trauma-focused Cognitive Therapy (bTF-CT), merging internet-modules with a few therapy sessions, may be a pathway to enhance treatment access while maintaining the benefits of face-to-face therapy.

**Objectives:**

This study aimed to evaluate the feasibility, acceptability, and preliminary effectiveness of bTF-CT for PTSD in routine care, as well as the feasibility of assessments and data collection.

**Method:**

A single-arm design was adopted. bTF-CT was provided to 17 participants across two psychiatric and one primary care clinic. Assessments were conducted pre, during, post and 6-months following treatment. We assessed feasibility and acceptability via self-report questionnaires, retention, and attrition rates. To estimate preliminary treatment effectiveness the PTSD Symptom Checklist (PCL-5) was used to assess PTSD symptom severity.

**Results:**

Treatment satisfaction was high with a mean score of 28.7 out of 32 on the Client Satisfaction Questionnaire (SD = 3.5). The dropout rate was low, with 88% treatment retention. Program adherence was satisfactory, with scores ranging from 2.13 to 3.13 out of 4 on the internet intervention patient adherence scale. On the PCL-5, 88% made a reliable change, 64% demonstrated a clinically significant change, and the mean change from pre to post was 24 points (*d* = 2.13). Some negative effects were reported, such as unpleasant memories, feelings, and disrupted sleep, but these were temporary according to symptom scales.

**Conclusions:**

bTF-CT appears to be acceptable, feasible, and potentially effective when delivered in routine care. A large-scale non-inferiority trial to assess effectiveness compared to a gold-standard treatment is warranted.

**Clinical Trial Registration:**

Clinicaltrials.gov, identifier NCT04881643.

## Highlights

- Blended trauma-focused cognitive therapy (bTF-CT) for PTSD, a fusion of internet-delivered and in-person therapy, was found to be acceptable, feasible and potentially effective for patients with PTSD in routine primary and secondary care.
- bTF-CT yielded high treatment satisfaction and low drop-out rates.
- Large reductions in symptoms of PTSD and depression were obtained, with effects maintained at a six-month follow-up.

## Introduction

Psychological therapy, in particular trauma-focused cognitive behavioural treatments (TF-CBT), are effective (1) first-line treatments of post-traumatic stress disorder (PTSD) (2, 3) and have demonstrated effectiveness also when implemented in routine care (4, 5). Yet, several factors seem to impede access to these treatments including long waitlists to specialist services, and a lack of trained clinicians (6, 7). Moreover, recipient factors such as scepticism about treatment effectiveness, practical obstacles, and fears of stigma and shame often inhibit treatment-seeking among PTSD-sufferers (8). Hence, there is a critical need for resource-efficient treatments for PTSD that are both effective and acceptable to patients, and that can be adapted to the needs and constraints of routine care services.

Internet-delivered CBT (I-CBT) for PTSD has been proposed to address these barriers, and while promising, results have been mixed (9). Several meta-analyses have examined the efficacy of I-CBT for PTSD, showing moderate to large effect sizes compared to passive and active non-trauma-focused controls, although with smaller effects than those seen in studies of standard TF-CBT (10–12). Moreover, retention rates for I-CBT in these studies were lower compared to controls with dropout rates from 25% (11) to 36% (10). However, a few recent studies of guided I-CBT for PTSD, in which self-help material is combined with regular but brief therapist guidance, have shown more promising results. Bisson et al. (13) found that their guided I-CBT treatment Spring was non-inferior to standard TF-CBT for patients with mild-moderate PTSD from a single trauma. Ehlers et al. (14) developed a guided internet-based version of cognitive therapy for PTSD (25, CT-PTSD), which was found to be superior to internet-based stress management therapy. These results were replicated with routine clinicians in a further study in UK primary care services (15). The effects for both Spring and internet-based cognitive therapy (iCT-PTSD) were large and comparable to outcomes found in studies of standard TF-CBT (14). both studies demonstrated drop-out rates of below 10%.

In sum, several versions of guided I-CBT for PTSD seem promising, with some programs demonstrating large to very large effects (13, 14). Yet, effect sizes and dropout rates vary, and the majority of studies have not been conducted in routine care with routine therapists and a full range of PTSD patients, including those with severe presentations commonly seen in clinical settings. Hence, while the evidence for I-CBT for PTSD is accumulating it remains inexhaustive.

We propose evaluating a blended treatment for PTSD, integrating I-CBT and in-person therapy. Blended therapy combines the accessibility, flexibility, and reduced therapist time of I-CBT (16), with the benefits of a few in-person sessions. The therapy sessions are added to support patients with more demanding trauma-focused interventions, such as approaching the trauma memory as well as individualizing treatment, and enhancing adherence. This approach may facilitate treatment engagement for patients preferring in-person therapy, potentially yielding treatment acceptability and effectiveness comparable to standard TF-CBT, while being more resource-effective. A few studies have compared blended CBT for other conditions to face-to-face CBT in routine care with good results (16, 17). Patients have also shown greater acceptability towards blended treatment compared to stand-alone internet treatments (18). Also therapists recognize the advantages of blended therapy in enhancing treatment efficiency but highlight challenges related to technological integration and limitations in terms of individualizing treatment (19).

This study is the first within a larger project aiming to evaluate a blended trauma-focused cognitive treatment (bTF-CT) developed for Swedish routine clinical care services. The treatment is based on Ehlers and Clark’s CT-PTSD treatment (20) with the accompanying internet-program adapted from the guided internet version of CT-PTSD (14).

The aim of this study was to explore acceptability, feasibility, and preliminary effectiveness of bTF-CT, including the feasibility of obtaining self-report measures pre, weekly, post and 6 months after treatment completion. Specific objectives were to: (i) investigate treatment satisfaction, credibility, and negative effects. (ii) estimate the completion rate of outcome measures (iii) explore patient adherence to treatment. (iv) investigate preliminary treatment effects on symptoms of PTSD, depression, anxiety, sleep, functionality, and quality of life; and (v) determine the proportion of patients meeting criteria for recovery and response. The overarching goal was to gather insights about the treatment and study procedures in preparation for a subsequent large-scale randomized controlled non-inferiority trial (RCT) to investigate the effectiveness of bTF-CT.

## Method

Given that this was the first study exploring bTF-CT in clinical settings an open-trial design was adopted. Randomization was not employed, as the main objective was to explore treatment acceptability, feasibility, and preliminary effectiveness. Self-report measures of symptoms, function and quality of life were administered pre, during, post, and at 6-months following end of treatment (FU-6). Feasibility data were collected prior to, during, and post treatment. The study was registered at Clinicaltrials.gov (NCT04881643) and approved by the Regional Ethical Review Board in Stockholm (Dnr 2020-07130).

### Procedure and setting

**Figure 1:**
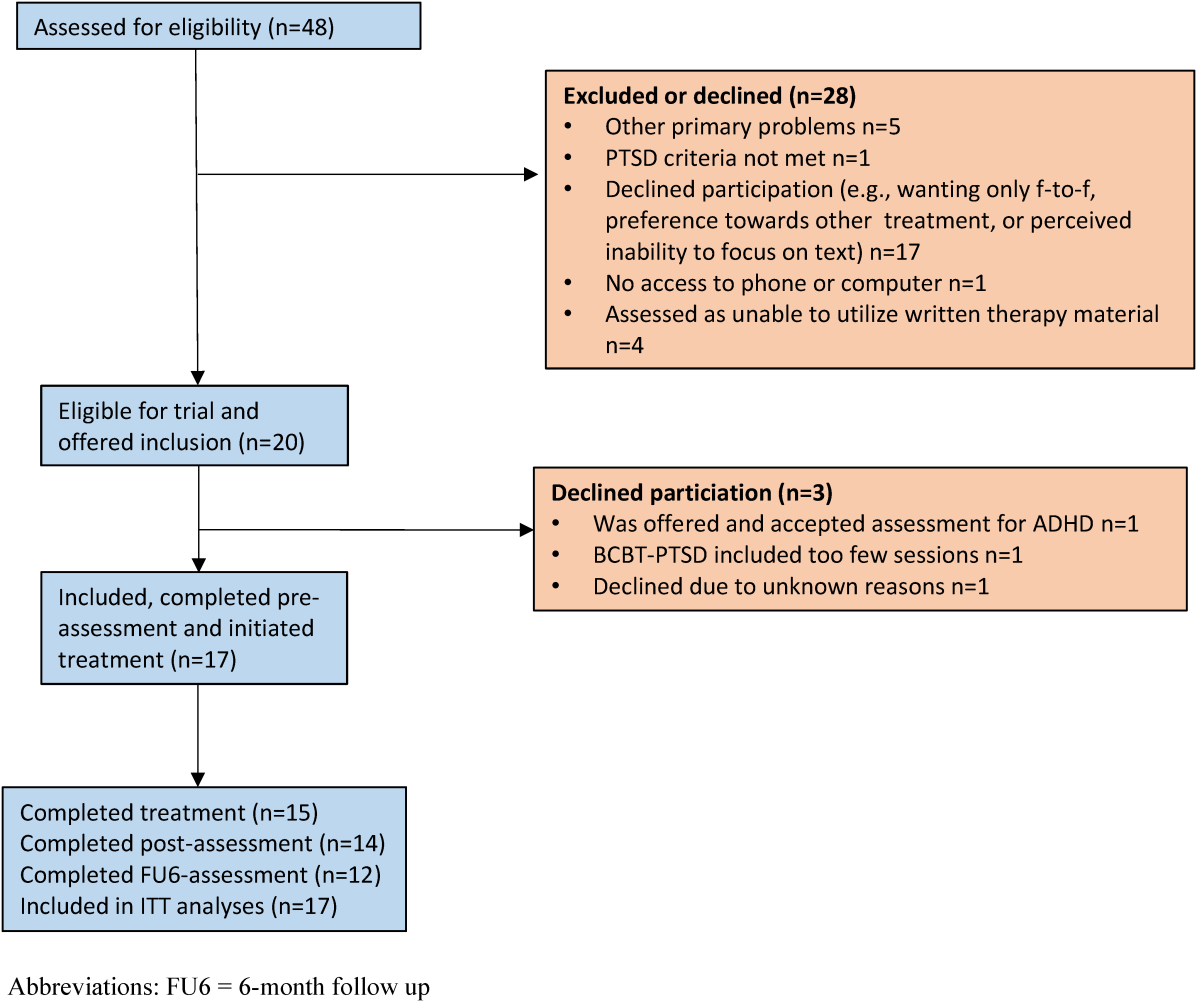
Participant flowchart.

The trial was conducted in two psychiatric outpatient clinics and one primary-care center in Stockholm, Sweden. In Swedish health care, mild to moderate PTSD is typically treated in primary care and moderate to severe PTSD in psychiatric clinics. Recruitment took place between May 2021 and April 2022, with the final post-treatment assessment completed in October 2022. Potential participants were identified from the regular flow of patients, or a waitlist of those with a provisional PTSD diagnosis following screening at the clinic. Patients were informed of the study and invited to an eligibility assessment including the MINI 7.0 (21) to assess psychiatric disorders. Interested patients completed screening on a secure, 2-factor authenticated website, with the Life Event Checklist (22), PCL-5 (23), and the PHQ-9 (24), and received written information about the study. Those wishing to proceed signed a consent form and, if they met eligibility criteria following the assessment, were included in the study. Excluded or declining patients were offered alternative treatment at the clinic. See **Figure 1** for the participant flow through the study.

### Eligibility criteria

To be included participants had to (a) have PTSD according to *DSM-5* diagnostic criteria as their principal problem (untreated bipolar, psychotic symptoms and high level of suicide risk were primary by default) (b) be able to read and write in Swedish, (c) not receive other psychological treatment during the trial (d), be minimum 18 years old, (e) not being under current trauma-related threat, (f) if on prescribed antidepressant medication, be on a stable dose for at least six weeks preceding treatment and (g) wish to partake in the study. Comorbid conditions (both independent and concurrent) were accepted given that they were assessed as secondary to PTSD. Eligibility criteria were chosen to represent typical patients with PTSD with a variation of PTSD severity, treated in primary and psychiatric care in Sweden.

### Participant characteristics

For a detailed depiction of demographic information see **Table 1**. The most common index trauma linked to PTSD was sexual assault. The majority had been exposed to more than one potentially traumatic event and had a comorbid psychiatric diagnosis. Five participants had previous experience of trauma-focused psychological treatment.

**Table 1:** Description of patients.

Variables		bTF-CT (*N*=17)
Gender	Women	15 (88%)
	Men	1 (6%)
	Non-binary	1 (6%)
Age	M (SD)	32 (11.9)
	Min-max	18-59
Occupational status	Working fulltime	7 (41%)
	Working part-time	4 (24%)
	Student (high school)	2 (12%)
	Student (university or equivalent)	3 (18%)
	Unemployed	1 (6%)
Highest education	Primary school	2 (12%)
	High/secondary school	6 (35%)
	University	9 (53%)
Main trauma linked to PTSD	Sexual assault/rape	6 (35%)
	Physical/psychological abuse in a relationship	5 (29%)
	Traumatic loss	2 (12%)
	Interpersonal violence	1 (6%)
	Acute physical illness	1 (6%)
	Traumatic birth	1 (6%)
	Sexual harassment/stalking	1 (6%)
Repeated or prolonged trauma		9 (53%)
Duration of PTSD*	Mean years (SD)	5,5 (5)
Previous experience of trauma-focused therapy	Yes	5 (29%)
Comorbid conditions according to MINI	Depression	7 (41%)
	Panic disorder	3 (18%)
	Insomnia	3 (18%)
	Social anxiety	1 (6%)
	ADHD	1 (6%)
	Eating disorder	1 (6%)
	GAD	1 (6%)

bTF-CT, Blended Trauma-Focused Cognitive Behaviour Therapy; PTSD, Post-traumatic stress disorder; GAD, Generalized Anxiety Disorder. MINI, The Mini International Diagnostic Interview. *N=13, reported duration of PTSD was absent in 4 assessments.

### Blended trauma-focused cognitive therapy

bTF-CT is a trauma-focused CBT delivered online and face-to-face. The treatment uses an internet-program containing self-study modules, which patients can access via their computer, tablet, or smartphone. In addition, six in-person therapy sessions are included. bTF-CT is based on Ehlers and Clark’s cognitive therapy for PTSD (25), one of the first-line TF-CBTs recommended in international guidelines (6, 7) and includes most of its core procedures (with the exception of site visits and some additional interventions for common cognitive themes). Our internet-program is a condensed adaptation of the internet-version of cognitive therapy for PTSD (14) but with fewer optional modules, videos, and case examples. Some modifications were made in the Swedish program (e.g. the order of the modules was more linear rather than adapted to an individual case formulation, there was more emphasis on telling the trauma story via narrative writing and/or imaginative exposure before updating hotspots and the reclaiming your life module did not include work on cognitive blocks to reclaiming activities). Dr Nick Grey from the Ehlers and Clark team supported the development of the Swedish program, and we were granted access to the iCT-PTSD modules and therapy materials which served as a template. However new texts, case examples, videos, illustrations, and work sheets were developed for the Swedish modules.

#### The program and modules

The program contains seven core modules covering key CT-PTSD procedures such as engaging with and updating trauma memories, modifying excessively negative appraisals about the trauma, identifying, and discriminating triggers and overcoming excessive fear appraisals through behavioural experiments (see **Table 2**). Additionally, three optional modules addressing guilt, shame, and rumination were adapted. The modules contain texts, written instructions, filmed illustrations, case examples, and worksheets, helping people address maintaining factors for their PTSD using CT-PTSD tools. For example, patients can write their trauma narrative and record behavioural experiments in the program. The modules are typically released by the therapists one or two at the time in the order in **Table 2**. Flexibility in order of release, tailored to individual presentation, is permitted.

**Table 2:** Means, and standard deviations for each item on the IIPASS rated by the clinician post-treatment.

IIPAS Item	1) Pace of work *Is the patient in phase with the treatment?*	2) Involvement in exercises *To what extent does the patient invest time and is actively involved in the exercises that are presented in the treatments*	3) Communication with the treatment provider *To what extent is the patient involved in communication with the therapist, responds to messages and takes initiative in topics of discussion and/or asking questions*	4) Motivation for change *To what extent is the patient willing to actively try and use the strategies presented in the treatment?*	5) Login frequency *How often is the patient active in the internet treatment?*
**Scale range**	0 = Inactive 4 = Completely in phase	0 = No exercises 4 = Has done all exercises, responded with interest and involvement	0 = Does not respond 4 = Ongoing dialogue with therapist, initiates communication	0 = Don’t use strategies 4 = Works actively and regularly on presented strategies	0 = Not active 4 = Often
	M (SD) 2.53 (1.24)	M (SD) 2.60 (1.18)	M (SD) 2.40 (1.06)	M (SD) 3.13 (1.13)	M (SD) 2.13 (0.99)

#### Therapy contacts and treatment structure

**Figure 2:**
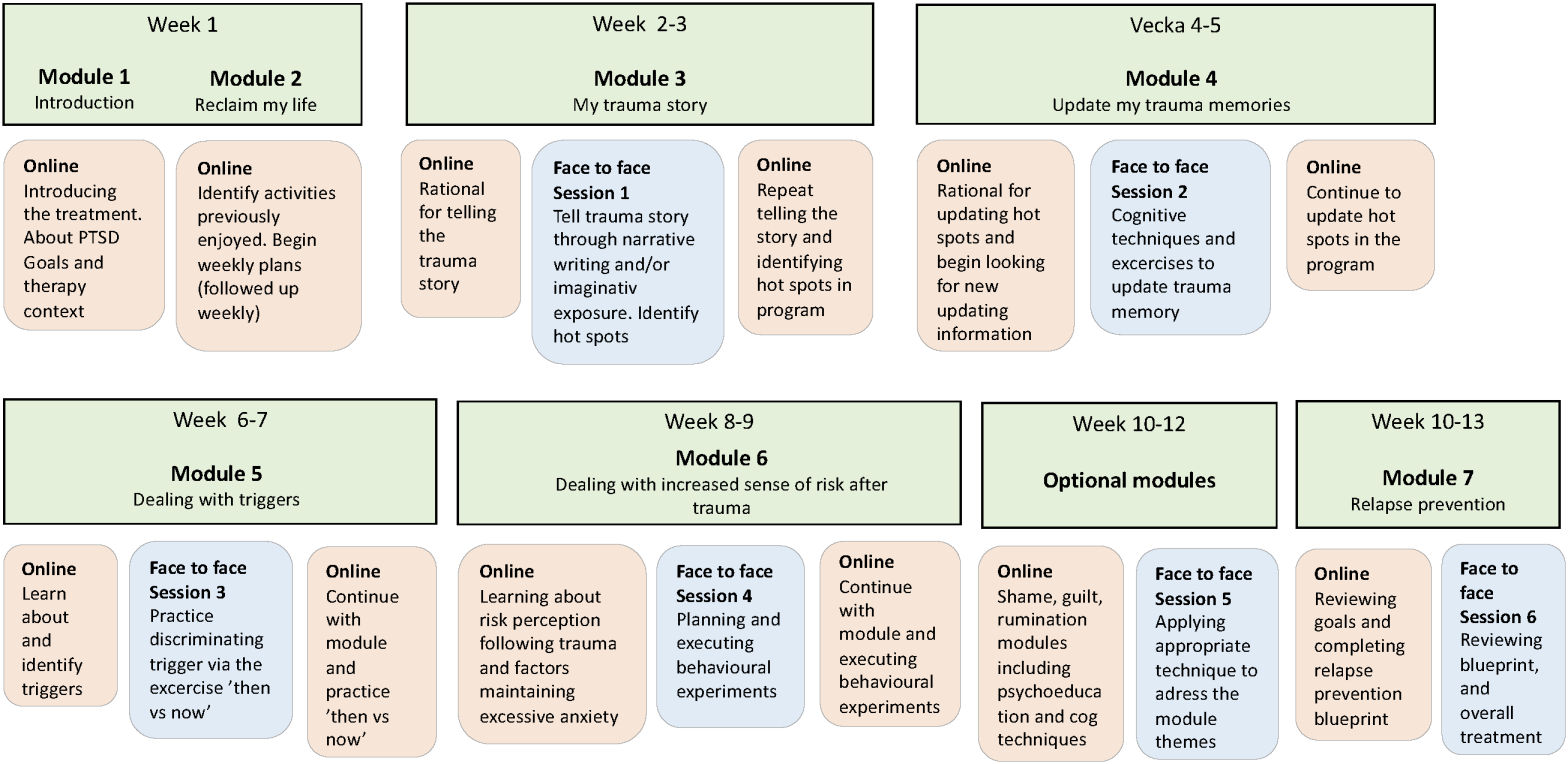
Prototype treatment structure.

Five of the core modules are accompanied by an in-person therapy session to support patients carrying out the key interventions included in the program modules. For example, in the session linked to the discriminating triggers module, the therapist and patient practice the ‘then vs now’ trigger discrimination technique, which the patient then continues with via the program. Sessions include discussing weekly measures, troubleshooting lack of program activity if needed and following up key exercises. Between sessions patients and therapists can communicate via a secure messaging facility in the program. A prototype timeline is introduced to patients at the start of therapy, outlining module and session flow. Patients complete two introductory modules in the first week online. For the remaining treatment patients spend two weeks per module and have therapy sessions fortnightly. Sessions are laid out in the timeline so that they align with completion of half the module. However, patients were informed that the timeline and structure was flexible and could be tailored to the patient’s pace and needs. For an overview of the treatment structure, see **Figure 2**.

### Therapists and supervisors

Seven licensed psychologists with experience of trauma-focused CBT received training in bTF-CT and delivered treatment within the study. Five were regular members of staff at the psychiatric clinics where the trial was conducted. Two, JL and SS, were from the research group who met patients in the primary care clinic. Supervision was conducted fortnightly by JL who is the lead author of this bTF-CT-program. In addition, supervision on supervision was given roughly once every two months by NG, expert in cognitive therapy for PTSD and consultant on this project.

### Measures

#### Feasibility and acceptability

Feasibility and acceptability was measured according to published guidelines (26, 27) albeit with an emphasis on the intervention as this was the first study to evaluate this bTF-CT. Feasibility refers to practical aspects of the treatment e.g. whether it can be carried out within a person’s day to day life. Acceptability refers to the extent to which the treatment was perceived as agreeable, tolerable, and satisfactory by patients. Feasibility was assessed via a clinician-rated adherence scale and acceptability via self-report questionnaires. Treatment dropout, defined as participants discontinuing, or completing less than 50% of the treatment (3 modules and 3 sessions), was considered an indicator of both acceptability and feasibility. A study of the dose-response curve in CBT for anxiety disorders found that among patients who achieved reliable and clinical significant change, 5 and 8 sessions out of a mean of 15 sessions was required for the respective outcome (28). Therapists documented time spent on sessions, messages, and phone calls in a case report form. Finally, the assessment and data collection procedure was assessed by calculating attrition of self-report measures. Instruments administered were:

The Client Satisfaction Questionnaire-8 (CSQ-8) includes 8 items (scale range 8 to 32) that cover different facets of treatment satisfaction. Example of items are *How satisfied are you with the amount of help you received* and, *to what extent has our program met your needs.* The CSQ-8 has demonstrated good psychometric properties including high reliability and construct validity and was administered post-treatment (29).

The 6-item Credibility and Expectancy Questionnaire (CEQ) (30) measures credibility (items 1-3) and expectancy (items 4-6) on a 1-9 or 0%-100% scale and was administered between weeks 1 and 3 of treatment. The CEQ has demonstrated sound psychometric properties.

The Negative Effects Questionnaire (NEQ) is a self-report instrument containing 32 items representing adverse or unwanted events during treatment, rated on a 5-point Likert scale ranging from “not at all” to “extremely so” with a factor structure of six: symptoms, quality, dependency, stigma, hopelessness, and failure. The NEQ has been validated as a reliable instrument (31) and was administered post-treatment.

The Internet Intervention Patient Adherence Scale (IIPAS) is a clinician-rated 5-item scale regarding patient adherence to internet therapy (32). Each item is rated on a 0-4 Likert scale with a total score ranging from 0-20 and was analyzed separately to explore the different areas of adherence covered by the instrument. IIPAS has shown good psychometric properties and was completed by the clinician post-treatment (32).

#### Preliminary effectiveness

Standardized validated self-report instruments measuring symptoms of PTSD, depression, anxiety, sleep, functionality, and quality of life were administered to investigate potential effectiveness. The primary endpoint was the post-treatment assessment (10-15 weeks following treatment initiation). We used the PTSD Checklist for DSM-5 (PCL-5) to assess PTSD symptoms, a self-report instrument with a symptom severity score ranging from 0-80 (27). PCL-5 has high internal consistency (alpha = 0.95) and good test-retest reliability (33).

Additional outcome measures were the following well-established and psychometrically sound instruments PHQ-9 (24) to assess depression, GAD-7 (34) to assess general anxiety, World Health Organization Disability Assessment (WHODAS; 35) to measure functional impairment, the Insomnia Severity Index (ISI; 36) to assess sleep disturbance and Brunnsviken Brief Quality of Life Scale (BBQ; 37) to assess quality of life. The PCL-5 and PHQ-9 was administered pre, weekly, post-treatment and FU-6. The other scales were completed pre/post-treatment and FU-6.

### Statistical analysis

#### Treatment effects

Statistical analyses were conducted using SPSS 29.0 and Jamovi 2.3. To estimate treatment effect on symptom measures, intention-to-treat linear mixed regression models were conducted. These models were utilized as they are advantageous in dealing with repeated measures and account for within-subject variability and missing data. After comparing various covariate structures (e.g. compound symmetry, autoregressive and unstructured) using Akaike’s Information Criteria, we opted for a compound symmetry structure since it fitted the data better than the competing covariate structures. The compound symmetry structure also estimates fewer parameters and is usually suitable for small samples. Statistical significance was set at 5%. Effect sizes (Cohens’ *d)* were calculated with 95% confidence intervals based on estimated means and pooled standard deviations (SD) from the mixed models. Notably, with such a small sample, outcomes will be interpreted with caution.

#### Response and remission

A reliable change index of 13.84 on the PCL-5 was calculated according to the criteria established by Jacobson and Truax (1991) with the following parameters included: a test-retest reliability of.84 (33), standard error of measurement (5), standard error of difference (7.07), and the standard deviation of the pre-treatment PCL-5 (12.5). Thus, a reduction on the PCL-5 of at least 14 points was classified as a treatment response. Clinically significant change (CSC) was defined as a reliable change as well as a post-treatment score under cut-off on the PCL-5. A cut-off value of 31 was chosen based on previous research and the International Society of Traumatic Stress Studies (ISTSS) guidelines recommending a PCL-5 cut-off of 31-33 (33, 38).

## Results

### Treatment retention, adherence and time spent on treatment

Fifteen participants (88%) completed the treatment, attending all six sessions and completing a minimum of three modules. The two participants who dropped out informed their therapists that they wished to discontinue, completing no more than two modules and attending one and two sessions, respectively. Three patients received 1-3 additional sessions due to severe panic attacks, difficult dissociation, or crisis management. Adherence was assessed using the IIPASS, with means and standard deviations for each item presented in **Table 2**. Therapists recorded an average total time of 7.7 hours (SD 2.3) per participant, with 6.9 hours (SD 1.9) spent on sessions and 0.7 hours (SD 0.8) on messages and phone calls.

### Data attrition

All participants completed pre-assessment, fourteen participants (82%) completed post-assessments and twelve participants (71%) completed FU-6. On average participants filled out 6/12 weekly assessments (M= 5.9 (*SD 3.6)*.

### Treatment satisfaction, credibility and expectancy, and negative effects

Fourteen participants completed the CSQ-8, with a mean score of 28.7 (SD 3.5) on a scale of 0-32, indicating high overall treatment satisfaction (29 on the CSQ corresponds with a response of 4, *very satisfied* on the majority of item and 3, *mostly satisfied* on some items). The CEQ scores suggested high treatment credibility (M = 7.19, SD = 1.12) and expectancy (M = 6.33, SD = 1.6). According to the NEQ, some negative effects were reported: 64% experienced unpleasant memories resurfacing, 69% reported more unpleasant feelings, and 35% felt sadder. Mean scores on these items ranged from 1.5 to 2, indicating a slight to moderate impact on well-being. Additionally, 29% reported increased sleep problems due to the treatment, with a mean score of 2.8, indicating a moderate to severe impact. Notably, the four participants reporting sleep issues did not show significant changes on the ISI (three improved by 1 point, and one worsened by 1 point from pre- to post-treatment).

### Treatment effects on clinical outcomes

Estimated means, standard deviations and effect sizes for all symptom measures are presented in **Table 3**. Initial analyses examined changes on the PCL-5 from pre-post treatment across 12 weekly assessment points and from post-treatment to FU6. Mixed models showed a significant effect of time on the PCL-5 from pre-post treatment (F = 17.32, *p* <.001) and no significant effect of time post-treatment to FU-6 (F = 0.38, *p* >.547). Estimated within-group effect sizes were large from pre to post treatment (*d =* 2.14) and negligible from post-treatment to FU6 (*d =* 0.12).

**Table 3:** Estimated means, standard deviations and effect sizes on all outcome measures from baseline to post treatment and post-treatment to FU6.

				PRE-POST			POST-FU6
Measure	Pre M (SD)	Post M (SD)	6MFU M (SD)	p-value	Cohen’s *d* (95% CI)	p-value	Cohen’s *d* (95% CI)
PCL-5	44.4 (11.0)	20.4 (11.4)	21.8 (11.8)	<.001	2.13 (1.39-3.16)	.547	0.12 (-0.57-0.83)
PHQ-9	14.8 (5.6)	7.8 (5.9)	8.9 (6.0)	<.001	1.19 (0.51-2.03)	.480	0.18 (-0.51-0.89)
GAD-7	10.9 (4.1)	6.1 (4.4)	6.1 (5.0)	<.001	1.10 (0.43-1.93)	.942	0.01 (-0.69-0.71)
ISI	16.2 (6.4)	13.4 (6.8)	11.8 (7.1)	.010	0.38 (-0.29-1.12)	.380	0.22 (-0.46-0.94)
WHODAS	35.5 (19.5)	22.9 (20.2)	25.3 (21.1)	.002	0.62 (-0.05-1.38)	.500	0.11 (-0.58-0.82)
BBQ	30.0 (8.4)	34.5 (8.9)	34.0 (9.2)	.014	0.51 (-0.17-1.25)	.860	0.05 (-0.64-0.76)

Pre, pre-treatment; Post, post-treatment; FU6, six-month follow-up; PCL-5, PTSD Checklist for DSM-5; PHQ-9, Patient Health Questionnaire; GAD-7, Generalized Anxiety Disorder assessment; ISI, Insomnia Severity Inventory; WHODAS, World Health Organisation Disability Assessment; BBQ, Brunnsviken Brief Quality of Life Inventory.

Treatment effects on the PHQ-9 and GAD-7 were analyzed from pre-post treatment including a mid-treatment assessment (week 6) and from post-treatment to FU6. Analyses of the PHQ-9 showed a significant effect of time from pre-post (F = 17.37, *p* <.001) and no significant effect from post-treatment to FU6 (F = 0.53, *p* >.480). Estimated within group effect sizes were large from pre-post (*d =* 1.2) and negligible from post-treatment to FU6 (*d* = 0.18). Further analyses showed significant effect of time on the GAD-7 from pre-post (F = 11.60, *p* <.001) but not from post-treatment to FU6 (F = 0.006, *p* >.942). Estimated within-group effect sizes were large from pre to post treatment (*d =* 1.12) and negligible from post-treatment to FU6 (*d =* 0.01). The descriptive statistics (marginal means and standard deviations) for the PCL-5 across all assessment points are displayed in **Supplementary Figure S1**.

Finally, effects on the additional measures (ISI, WHODAS and BBQ) were analyzed from pre-post treatment and from post-treatment to FU6. The analyses demonstrated a significant effect of time from pre-post on WHODAS (F = 14.32, *p =* 0.02), BBQ (F = 7.97, *p* = .014) and ISI (F = 8.68, *p* = .01). There was no significant effect of time from post-treatment to FU6 on WHODAS (F = .49, *p* >.5), BBQ (F = .31, *p* >.86) and ISI (F = .82, *p* >.38). Estimated effect sizes pre-post were small to moderate for ISI, WHODAS and BBQ (*d* = 0.38 - 0.62) and small to negligible post-treatment to FU6 (*d* = 0.05 – 0.22).

### Treatment response and remission

Fifteen participants (88%) met criteria for reliable change, e.g. improved by a minimum of 14 points on the PCL-5, including two who did not complete the post-treatment assessment. For these two participants, the last observation carried forward (LOCF) method was applied—one completed the treatment and provided a final weekly assessment just before the last session (week 13), and the other dropped out mid-treatment (final assessment week 6). Three patients were excluded from the clinically significant change calculation (CSQ) due to pre-treatment scores below the cut-off value of 31. Among the remaining 14 patients, 9 (64%) met criteria for CSC, achieving both reliable change and post-treatment scores below 31.

## Discussion

The aim of this study was to explore feasibility, acceptability, and preliminary effectiveness of a novel blended trauma-focused cognitive therapy for PTSD in routine care, as well as the feasibility of the study’s assessment and data collection procedures. Our results indicate that patients found bTF-CT acceptable and feasible, treatment retention and adherence was high and assessment completion satisfactory. A few negative effects were reported. Treatment effects on symptoms of PTSD, depression, and anxiety were large, while effects on sleep, functionality and quality of life were small to moderate.

Our small sample (17 participants), appears to be fairly representative in terms of patient characteristics such as type of trauma, number of traumatic experiences, and psychiatric comorbidity (Kessler) (39). Of note is that 88% of the sample were female. However, in clinical PTSD-studies with non-veteran populations, the majority of participants have typically been female (60-80%) (40). Likely reasons are that PTSD is more common among women than men, and women are more likely to be exposed to sexual assault (40), which was the most reported index trauma in this study.

Patient satisfaction was high among treatment completers, as indicated by the CSQ-8 (*M =* 28.7/32). This outcome is in line with, or surpasses, previous studies of I-CBT for PTSD and is similar to traditional TF-CBT. Bisson et al. (19) reported that in their SPRING study patients in the I-CBT group scored 26.9 on the CSQ-8 while patients in the standard TF-CBT group scored 29.8. Additionally, a recent study of online prolonged exposure set in psychiatric care, showed an average CSQ-8 score of 22 (41). A noteworthy shortcoming of the CSQ-8 is that usually only completers of the post-assessment fill out the questionnaire, which in our study was 82%. This indicates that the picture of treatment satisfaction could be somewhat optimistic. Further, patient scores on the CEQ were in line with previous studies investigating treatment credibility and expectancy for PTSD and other conditions (30, 42). While we did not explore associations with outcome in this study, these findings indicate that the majority saw bTF-CT as credible and expected to improve.

We also examined treatment retention as an acceptability and feasibility marker, finding that 12% of patients dropped out, which aligns with the more favorable range of dropout rates reported in studies of I-CBT for PTSD (iCT-PTSD; 14), and TF-CBT (43). A meta-analysis found the pooled dropout rate of the various TF-CBTs to be 18% whereas meta-analyses of I-CBT for PTSD have reported dropouts of 26-36% (10, 11, 43). The relatively low dropout rate in our study could be due to various factors. Evidently, when dealing with a small sample, it could be due to chance. Another possible factor is the cognitive therapy approach underpinning bTF-CT. Studies have repeatedly demonstrated relatively low dropouts for cognitive therapy for PTSD, including a dissemination study in routine care with 330 patients where 86% completed treatment (4, 43). The blended format may also have promoted treatment retention. A qualitative study would likely shed more light on these queries and would be a welcome next step in assessing acceptability of bTF-CT.

The blended treatment protocol appeared feasible in terms of treatment adherence and retention. With regards to the treatment structure and proposed order of modules and sessions, our impression, based on verbal reports from the therapists, is that this framework rarely crystalized and typically more flexibility was required. A flexible formulation-driven approach is in line with the CT-PTSD method and may be a treatment feature to adjust for future studies. This will be further explored in an upcoming qualitative study conducted with patients and therapists from the current study. Therapist-rated adherence was moderate to good, with high session attendance and satisfactory engagement with the program. The adherence item that received the lowest mean score (2.13/4) was *login frequency* and the item that received the highest mean score (3.13/4) was *motivation for change*. Interestingly, Lenhard et al. (36) found that items on the IIPASS of structural nature such as login frequency did not corelate with treatment outcome, whereas items associated to therapy activity such as *motivation for change* did.

*Unpleasant memories* and *feelings* were the most common negative effects reported after treatment, but with mild-moderate impact on well-being. Affected sleep was reported by a minority of patients (29%) albeit with a moderate to large impact on well-being. Notably, in terms of negative effects, it is unclear whether participants reported negative consequences of the treatment or temporary challenging experiences that are part of many psychological treatments. Indeed, a limitation of the NEQ is that it does not distinguish between temporary and lasting negative effects, and future studies are needed to clarify the distinction. Some distress is embedded in TF-CBT, partly due to the nature of approaching painful memories and avoided stimuli (43). The large effects observed on the PCL-5 and PHQ-9 in this study suggests that it is unlikely that the above negative effects were long-lasting.

Our results showed large improvements in PTSD symptoms (*d =* 1.93 – 2.13*).* Patients made a reliable improvement with 88% meeting criteria for reliable change and 64% for clinically significant change on the PCL-5. These outcomes are promising and fit well with previous findings. Two meta-analyses investigating effectiveness of TF-CBT in routine care reported effect sizes of 1.75-2.59 (5, 44), and a large dissemination study of CT-PTSD found reliable change and clinical significant change rates of 79% and 57% respectively (4). This provides tentative support for the notion that the blended format may yield treatment effects similar to those found in in-person TF-CBT studies when implemented in routine care.

Our results also showed large effects on measures of depression and anxiety and small to moderate changes on functionality, sleep, and quality of life. Again, these outcomes are in line with studies of in-person TF-CBT and somewhat larger than those reported in a meta-analysis of I-CBT (5, 10). The small to moderate effect on the sleep measure ISI (*d* = 0.38 - 0.66) is of note since 29% also reported a moderate to large negative treatment effect on sleep. In contrast one study showed that CT-PTSD led to significant improvement of self-rated sleep duration and quality compared to a non-trauma-focused therapy with a pre- to-post treatment effect size of 0.86 – 1.05 on the ISI (45). The reasons for the mediocre response on sleep rates in this study is unclear. It may be due to limited scope to address sleep difficulties in this blended treatment. It could also indicate suboptimal treatment effect on sleep disturbing symptoms such as reexperiencing and hypervigilance, although those symptoms did improve according to the PCL-5 with a large effect size. Finally, the broad variation in pre-treatment scores on the ISI may also account for the blunt outcomes.

In terms of data attrition 82% and 71% completed post and FU-6 assessment respectively which is in line with, or better than, other studies (5). However, in the literature attrition often refers to dropout whereas we distinguish treatment discontinuation from not completing an assessment. Hence comparison to other studies can be somewhat blunt. Notably, on average, participants completed only half of the weekly assessments. Reasons for this are unclear but may include technical issues (e.g. difficulties logging in to the data platform), insufficient information at inclusion, or too many questionnaires. Obtaining weekly measures is advantageous and has clinical utility as well as research benefits as repeated measures allow for more robust and precise data analysis models.

Finally, therapists reported spending an average of 7.7 hours on bTF-CT per patient (including sessions, phone calls and messages). In contrast, prolonged exposure, one of the most adopted TF-CBT protocols constitutes of 8-15 90-minute sessions ranging from 12-22.5 hours. Hence, there are good reasons to hypothesize that this treatment could be more time-efficient, and potentially more cost-effective than standard in-person PE and other TF-CBT treatments. Of course, these assumptions would need to be empirically examined. Importantly, a few studies have shown that PE can successfully be delivered with 60-minute sessions (46). Also, other brief versions of TF-CBT such as Written Exposure Therapy have shown to been effective for PTSD (47). Thus, bTF-CT may be one of various brief and effective PTSD treatments.

### Strengths and limitations

A key strength of this study is its implementation in clinical settings using routine therapists as well as including patients representing the full range of PTSD severity, enhancing the ecological validity. The broad array of feasibility and acceptability measures provides a comprehensive picture of treatment reception, informing both treatment development and subsequent studies. Further, the clear structure of the internet program, alongside regular clinical supervision and training, likely contributed to high treatment fidelity and overall quality of the intervention. However this could also be viewed as a limitation to the study’s ecological validity as it may not reflect standard practice in routine care, potentially limiting the generalizability of the findings. The study also has several limitations. First, we make assumption regarding acceptability and feasibility based on self-report measures and retention. Dropouts as a proxy measure for acceptability has been questioned as it can implicate other things such as early improvements (48). Also, the therapist perspective was not included in this study, which is important for assessing treatment feasibility. A more complete picture of acceptability and feasibility may have been achieved by including qualitative data and analysis. Interviews with patients and therapists were conducted but, to not dilute important outcomes, these findings will be reported separately. Further, the small sample size and absence of a control group limit the ability to distinguish the specific effects of the intervention from other external factors, highlighting the importance of cautious interpretation of the results (49).

### Implications for future research and clinical utility

Patients in this study appeared to respond well to bTF-CT and promising improvements were obtained in terms of PTSD-symptom reduction. Dropout rates were low, and self-reported acceptability high. This offers tentative support to the notion that bTF-CT could be a viable treatment option in routine clinical settings potentially promoting and improving access to treatment for patients with PTSD. Identified areas for improvement were: A) Treatment structure and content, where more flexibility and specifically targeting sleep difficulties in the program may be beneficial, B) Adherence to weekly measures possibly achieved via fewer questionnaires, or clearer information at study inclusion, and C) Adherence to the internet program. We will take steps towards calibrating these features of the treatment and procedures in preparation of subsequent studies.

## Conclusions

bTF-CBT appears to be acceptable, feasible, and potentially effective when delivered in routine care. A large-scale non-inferiority trial to assess effectiveness compared to a gold-standard treatment is warranted.

## Data Availability

With respect to the legal framework regulating access to research data in Sweden and the European Union, the data are not freely accessible due to regulations regarding personal integrity in research, public access, and privacy; each request for data must therefore be assessed by the Swedish Ethical Review Authority. Data access can be given after this assessment. Requests to access the datasets should be directed to johan.lundin.1@ki.se.
