# Supplementary material for: Integrating digital and in-person therapy for PTSD: feasibility and acceptability of blended trauma-focused cognitive therapy in routine care
Source: Front Psychiatry. 2024 Sep 5;15:1447651. doi: 10.3389/fpsyt.2024.1447651 (PMC11410639; doi:10.3389/fpsyt.2024.1447651)
Supplement: Supplementary file 1 [file Image1.pdf]

Figure S1

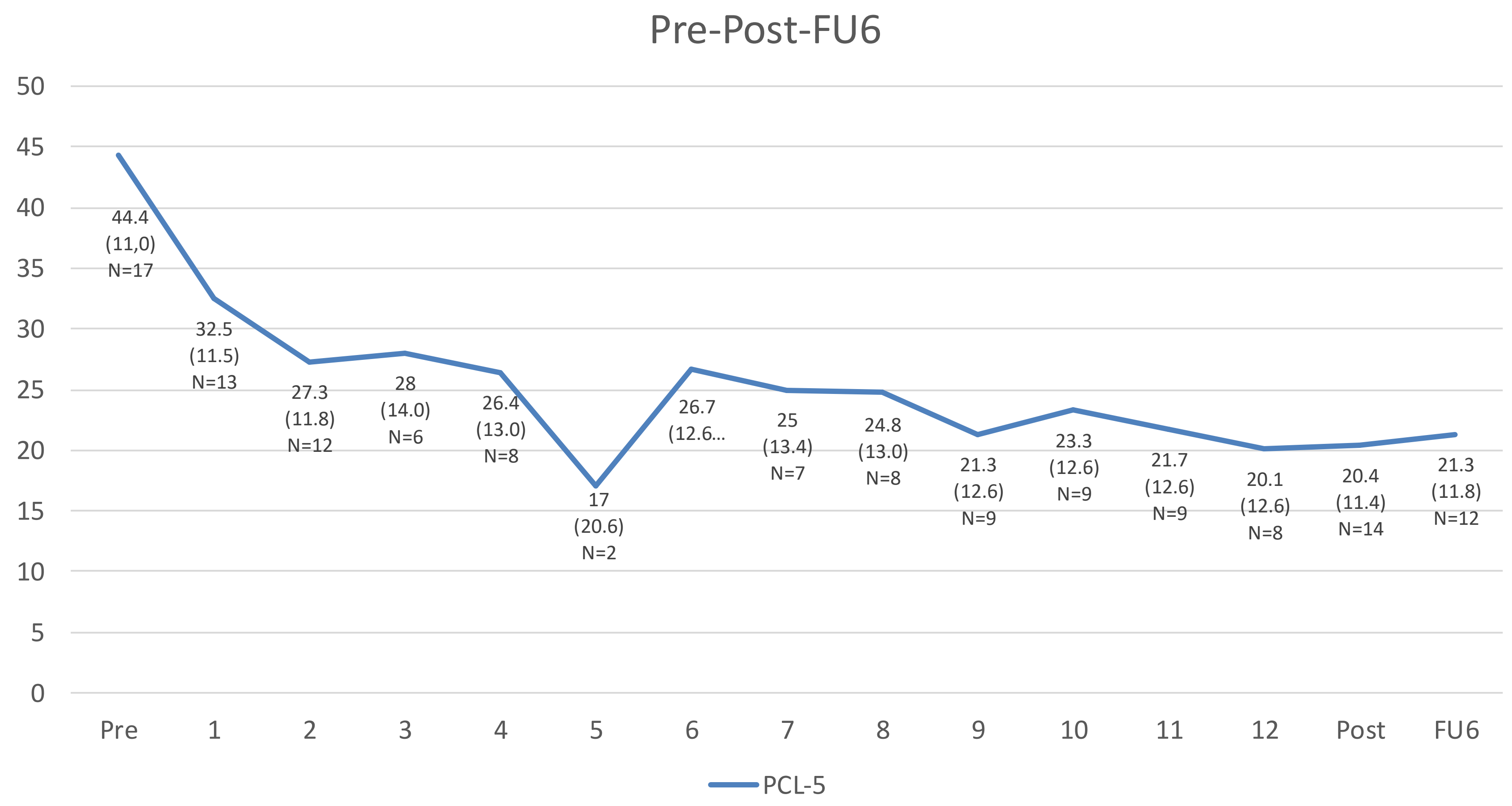

Figure X showing estimated marginal means, (and standard deviations) for the PCL-5 across all assessment-points from pre to FU-6. We agreed not to include this figure in the main manuscript as different participants filled out each weekly measure and therefore the figure may convey a somewhat skewed picture of the progress over time for the group.
